# Tryptophan metabolites and gut microbiota play an important role in pediatric migraine diagnosis

**DOI:** 10.1186/s10194-023-01708-9

**Published:** 2024-01-05

**Authors:** Junhui Liu, Kaiyan Xi, Linlin Zhang, Mugu Han, Qingran Wang, Xinjie Liu

**Affiliations:** 1 Department of Pediatrics, Qilu Hospital of Shandong University, No.107 West Wenhua Road, Jinan, 250012 Shandong Province China; 2 Shandong First Medical University Affiliated Provincial Hospital, Huaiyin Distinct, Jingwuweiliu Road, Jinan, 250021 Shandong Province China; 3 Qilu Hospital of Shandong University Dezhou Hospital, 1166 Dongfanghong West Road, Decheng District, Dezhou, 253000 Shandong Province China

**Keywords:** Pediatric migraine, Gut microbiota, Tryptophan metabolism, Diagnostic biomarkers

## Abstract

**Background:**

The pathogenesis of pediatric migraine remains unclear and presents challenges in diagnosis. Recently, growing evidence has indicated that the gut microbiota can exert modulatory functions at the gut-brain axis by directly or indirectly regulating tryptophan metabolism. Consequently, we aimed to elucidate the potential association among gut microbiota, tryptophan metabolism, and pediatric migraine while also identifying diagnostic biomarkers for pediatric migraine.

**Methods:**

The gut microbiota composition of 33 migraine children and 42 healthy children, aged less than ten years, from the GMrepo database, was analyzed using the Shannon index, Simpson index, principal coordinates analysis, and Wilcoxon rank-sum test. Microbial diagnostic biomarkers were identified using linear discriminant analysis effect size, ridge regression, and random forest. Plasma concentrations of tryptophan metabolites investigated by enzyme-linked immunosorbent assay were compared between 51 migraine children and 120 healthy children, aged less than eighteen years, using t tests and analysis of variance. The receiver operating characteristic curve was performed to evaluate the diagnostic value of microbial and metabolite biomarkers in pediatric migraine.

**Results:**

Differences in the composition of gut microbiota, notably the genera that regulate tryptophan metabolism, were observed in pediatric migraine children. Further investigations revealed a significant decrease in plasma kynurenic acid levels (*p* < 0.001) among migraine children, along with a significant increase in serotonin (*p* < 0.05) and quinolinic acid (*p* < 0.001). Subsequently, we established the normal reference intervals for plasma concentrations of tryptophan metabolites in children. More importantly, the ratio of kynurenic acid to quinolinic acid (AUC: 0.871, sensitivity: 86.3%, specificity: 83.3%) exhibited excellent diagnostic efficacy for pediatric migraine.

**Conclusion:**

Our study suggests that the gut microbiota may play an important role in the development of pediatric migraine by regulating tryptophan metabolism. We believe that microbial and metabolite biomarkers are sensitive diagnostic tests for pediatric migraine.

**Trial registration:**

The study was registered at ClinicalTrials.gov (NCT05969990).

**Supplementary Information:**

The online version contains supplementary material available at 10.1186/s10194-023-01708-9.

## Introduction

Migraine is a prevalent neurological disorder that affects approximately 10% of children. According to the Global Burden of Disease Study 2016, migraine ranks as the second leading cause of disability [1]. Migraine in children frequently co-occurs with functional gastrointestinal disorders. This interrelation highlights the important role of the gut-brain axis in migraine, which involves gut microbiota [2]. Previous research has indicated an association between gut microbiota dysbiosis and the development of certain neuropsychiatric disorders, such as autism spectrum disorders and depression [3, 4]. However, the role of the gut microbiota in pediatric migraine has not been investigated.

Tryptophan (Trp) is an essential amino acid metabolized in the human body in three main pathways: the serotonin (5-hydroxyptamine, 5-HT), kynurenine (KYN), and microbiota-related indole pathways [5]. Studies have indicated that metabolites produced in the serotonin and kynurenine pathways are related to migraine in adults [2, 5]. However, the role of tryptophan metabolites in pediatric migraine remains unexplored. Furthermore, accumulating evidence indicates that gut microbiota can exert modulatory functions in neuropsychiatric disorders, such as autism spectrum disorders and depression, by directly or indirectly affecting luminal tryptophan metabolism [4, 6]. Studies have found that gut microbiota can metabolize tryptophan, both through the serotonin pathway and the kynurenine pathway, resulting in the production of metabolites such as serotonin, kynurenine, kynurenic acid (KYNA), and quinolinic acid (QUIN) [7–9]. However, to date, no research has explored the relationship between gut microbiota, tryptophan metabolites and pediatric migraine.

Moreover, there are many challenges in the diagnosis of pediatric migraine. The diagnosis of pediatric migraine relies on clinical criteria [10]. However, the distinctive duration and localization of headaches in children compared to adults, coupled with the challenges young children face in accurately describing their symptoms, hinder the accurate diagnosis of pediatric migraine using traditional diagnostic classifications. Thus, there is an urgent need to identify objective, rapid, and quantitative biomarkers for the accurate diagnosis of pediatric migraine.

**Fig. 1:**
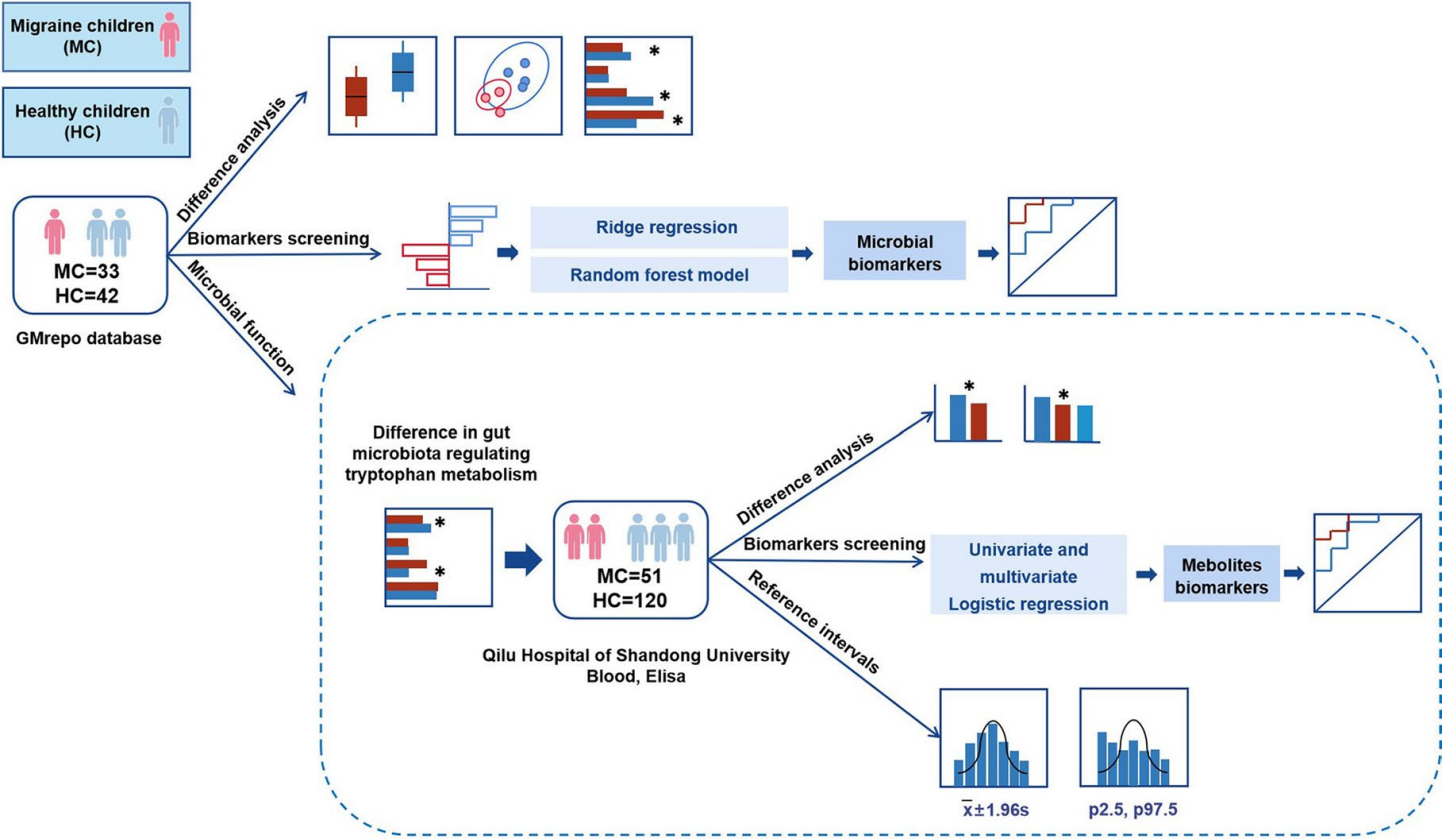
Flowchart

The primary objective of this study was to explore the pathophysiology of pediatric migraine and identify specific diagnostic biomarkers for pediatric migraine. First, we investigated alterations in the composition of the gut microbiota, particularly those involved in regulating tryptophan metabolism, in migraine children and identified specific microbial diagnostic biomarkers for pediatric migraine. Second, we analyzed the changes in the plasma concentrations of tryptophan metabolites in migraine children and identified specific metabolite biomarkers for diagnosing pediatric migraine. Eventually, we will establish normal reference intervals for tryptophan metabolites in children (Fig. 1).

## Materials and methods

### Study participants

Gut microbiota abundance of migraine and healthy children was provided by the American Gut Project [11] (PRJEB11419), housed in the GMrepo database (https://gmrepo.humangut.info/home). The investigation of plasma levels of tryptophan metabolites involved migraine and healthy children from Qilu Hospital of Shandong University. The inclusion criteria for migraine children delineated that boys were under the age of 10 and girls under the age of 9 for the gut microbiota study, and under the age of 18 for the tryptophan metabolite study. The criteria also included a body mass index (BMI) within the normal range for their age and gender, and a diagnosis of migraine with or without aura according to the International Classification of Headache Disorders, 3rd Edition. Exclusion criteria for migraine children comprised any drug use exceeding two months, secondary headaches, mental illnesses, congenital disorders, and other severe organ diseases. Healthy children were included if boys were below 10 years and girls below 9 years for the gut microbiota study, below 18 years for the tryptophan metabolites study, with a BMI within the normal range, and without any acute or chronic diseases, particularly neurological diseases. Additionally, they should not have used medication in the preceding two months.

### Procedures

Participants in our study about tryptophan metabolites had to complete a questionnaire that asked for their basic information and medical history. The questionnaire was completed by participants’ guardians if they were under the age of six. Before collecting blood, participants had to fast for eight hours and then rest in a seated position for ten minutes. Within eight hours of the start of the migraine, blood was drawn during the ictal stage. Blood was drawn during the interictal period with no migraine episodes for twenty-four hours before and after blood sample collection. Either the right or left antecubital vein was used to draw blood.

### Demographic and clinical characteristics

Data, such as age, sex, course, frequency, duration of attacks, visual analog scale (VAS) score, pain sites, accompanying symptoms, and aura of migraine, were gathered. The accompanying symptoms included nausea, vomiting, photophobia, and phonophobia. Flashing, bright spots, black spots, blurred vision, etc., are symptoms of visual aura. Pins and needles paresthesia as well as numbness on one side of the body, face, or tongue are symptoms of sensory aura.

### Measurements

Centrifugation was performed on the blood for 30 min at 4 °C and 3000 rpm (Hangzhou Allsheng Instruments Co., Ltd, Hangzhou, China). Until analysis, the supernatant was kept at -80 °C. Enzyme-linked immunosorbent assay kits (Jiangsu Meimian Co., Ltd, Jiangsu, China) were used to determine the plasma concentrations of five metabolites in the samples: TRP, 5-HT, KYN, KYNA, and QUIN. The detection threshold for TRP was 1.0 pg/ml, 5-HT was 1.0 ng/ml, KYN was 0.001 nmol/l, KYNA was 1.0 µmol/l, and QUIN was 1.0 nmol/l. The standards were introduced to respective standard wells in 50 µl of various concentrations. Then, sample wells were filled with 40 µl of sample diluent and 10 µl of sample. Per sample, a single sub-well was set up. The 96-well plates were filled with 100 µl of HRP, and they were then incubated at 37 °C for 60 min. Then, using the wash solution, the plates were washed five times. Next, 50 µl of substrate solution A and 50 µl of substrate solution B were added to each well. The 96-well plates were incubated for 15 min without any exposure to light. Then, using a microplate reader (Tecan Trading AG, Switzerland), the optical density was measured at 450 nm after adding 50 µl of stop solution to each well. Standards with known quantities of TRP, 5-HT, KYN, KYNA, and QUIN were used to generate an optical density curve.

### Statistical analysis

SPSS for Windows (version 27.0, SPSS Inc., Chicago, IL, USA) and R environment (v4.2.2) were used to conduct the statistical analysis. The Kolmogorov‒Smirnov test and Levene’s test were used to evaluate the normality and homogeneity of the data, respectively. Data are expressed as mean ± SD (standard deviation) for normal distribution and median ± IQR (Interquartile Range) for skewed distribution. It was considered statistically significant when both sides had *p* < 0.05.

In the study of gut microbiota, microbial taxa with a relative abundance > 0.1% in at least 50% of cows within each group were included in downstream analysis. The Wilcoxon rank-sum test was used to compare the differences in richness and diversity of gut microbiota in the two groups. Principal coordinates analysis (PCoA) and analysis of similarities (Adonis) with a permutation of 999 were used to assess intergroup differences. Ridge regression, random forest, and linear discriminant analysis effect size (LEfSe) were used to identify the specific genera that significantly differed migraine children from healthy children, with a threshold LDA score of > 2.0 (Tutools platform, a free online data analysis website, https://www.cloudtutu.com/). Receiver operating characteristic curve (ROC) were used to evaluate the diagnostic ability of the specific genera combination for diagnosing pediatric migraine. Spearman correlation was performed to assess the association among the gut microbiota.

In the study of tryptophan metabolites, the t-test was used when the data of tryptophan metabolites demonstrated normal distribution, and the Mann–Whitney test was used when the distribution deviated from normality to compare the differences of tryptophan metabolites concentrations between two groups. The differences between more than two groups were analyzed with Welch's ANOVA test. Logistic regression analysis and ROC analysis were used to evaluate the diagnostic value of tryptophan metabolites in pediatric migraine.

## Results

### Gut microbiota dysbiosis in pediatric migraine

#### Study population

We collected gut microbiota data from 33 migraine children (17 boys, mean age 7.48 ± 1.95 years, mean BMI 16.17 ± 3.09) and 42 healthy children (22 boys, mean age 7.57 ± 2.23 years, mean BMI 16.78 ± 1.84). There was no difference in the mean age, BMI, and sex between the migraine and healthy groups (Table 1).

**Table 1:** Demographic characteristics of participants in the study of gut microbiota

Demographic	Migraine (*n* = 33)	Healthy (*n* = 42)	*p*
Country	United States	United States	
Sample	Stool	Stool	
Age (years)	7.48 ± 1.95	7.57 ± 2.23	0.862
Gender (male)	17(51.5%)	22(52.4%)	0.838
BMI	16.17 ± 3.09	16.78 ± 1.84	0.335

#### Gut dysbiosis in children with migraine

**Fig. 2:**
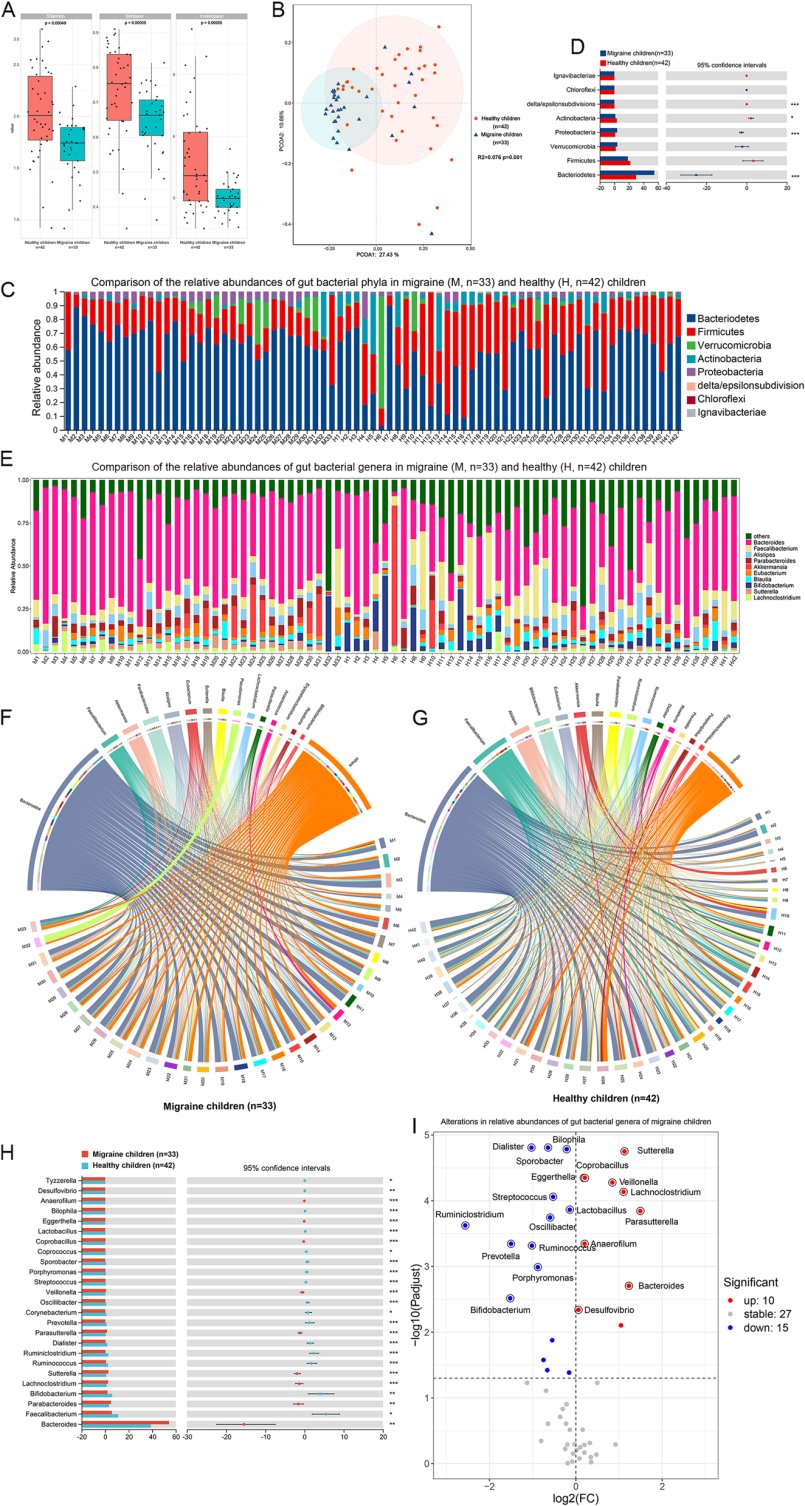
Gut dysbiosis in pediatric migraine. **A** Comparison of the α diversity of the gut microbiota in healthy children and migraine children by calculating Shannon, Simpson and Insimpson. **B** Plot of principal coordinates analysis (PCoA) based on the Bray‒Curtis distance matrix of the gut microbiota in healthy children and migraine children. **C** Histogram of the relative abundances of gut bacterial phyla in healthy children and migraine children. **D** Comparison of the relative abundances of gut bacterial phyla in healthy children and migraine children was used with the Mann–Whitney test. **E** Histogram of the relative abundances of gut bacterial genera in healthy children and migraine children. **F** The composition of gut bacterial genera in migraine children. **G** The composition of gut bacterial genera in healthy children. **H** Comparison of the relative abundances of gut bacterial genera in healthy children and migraine children was used with the Mann–Whitney test. **I** Volcano plot shows the alterations in the relative abundances of gut bacterial genera in migraine children

The alpha diversity of gut microbiota measured by the Shannon index and Simpson index (all *p* < 0.001, Fig. 2A) showed a significant decrease in gut microbiota richness and evenness of the migraine group compared to those of the healthy group. To assess the overall diversity of the gut microbiota between the two groups, PCoA analysis of the bray distance was performed and demonstrated significant differences between the migraine and healthy groups (R^2^ = 0.076, *p* = 0.001, Fig. 2B), indicating that gut microbial dysbiosis existed in children with migraine. At the phylum level, Bacteroidetes and Firmicutes were dominant in all groups (Fig. 2C). Interestingly, there were significantly higher levels of Bacteroidetes and Proteobacteria and lower levels of Actinobacteria and delta/epsilon subdivisions in the migraine group than in the healthy group (Fig. 2D).

At the genus level, Bacteroides, Faecalibacterium, and Alistipes were dominant in all groups (Fig. 2E). The gut microbiota composition of the migraine group was mainly composed of Bacteroides, Faecalibacterium, and Akkermansia (Fig. 2F). However, the healthy group was composed of Bacteroides, Faecalibacterium, and Alistipes (Fig. 2G). To identify differences at the genus level, the Wilcoxon rank-sum test was performed, showing the relative abundance of nine genera higher in the migraine group than in the healthy group. Furthermore, we found significantly lower levels of sixteen genera in the migraine group (Fig. 2H). To clearly show the change in the relative abundance of gut microbiota in migraine children compared with healthy children, we plotted a volcano plot with *p* < 0.05 and log2FC > 1 parameters, and the findings were in accordance with the results of the Wilcoxon rank-sum test (Fig. 2I). We also compare the difference of gut bacterial genera composition between migraine and healthy children in different sex groups (Supplementary Fig. 1), which indicates that the relative abundance of Bacteroides, Lachnoclostridium, Eggerthella, Coprobacillus, Bilophila, Anaerofilum, Parasutterella, Sporobacter were different between migraine and healthy children both in male and female group. The relative abundance of Dialister, Bifidobacterium, Porphyromonas, Sphingobacterium, Lactobacillus, Prevotella, Streptococcus, and Tyzzerella were different between the two groups in the female group, while in the male group, the relative abundance of Sutterella, Veillonella, Parabacteroides, Erysipelatoclostridium, Desulfovibrio, Dehalococcoides, and Ruminiclostridium differed in the two groups.

#### The gut microbiota-based signature can predict pediatric migraine

**Fig. 3:**
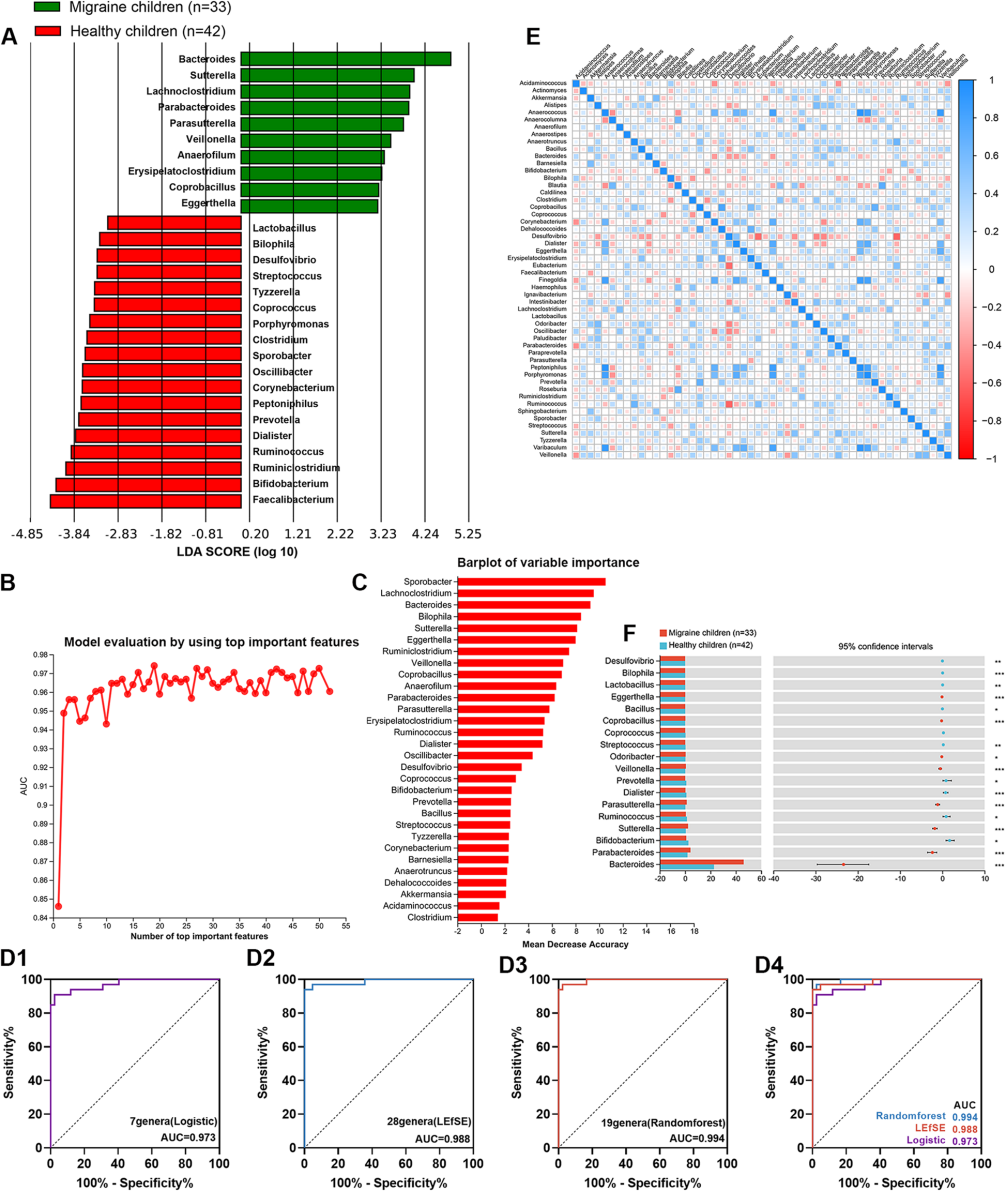
Exploration of diagnostic microbial biomarkers and the association between gut microbiota and tryptophan metabolism. **A** Linear discriminant analysis effect size (LEfSe) analysis for identifying diagnostic microbial biomarkers. **B** AUC validation method for random forest. The number of genera (*n* = 19) at the point with the highest AUC value was selected to construct the predictive diagnostic model. **C** Random forest for identifying diagnostic microbial biomarkers. The importance of genera decreases from the top to the bottom, and based on the evaluation results of the AUC method, the top 19 genera were selected to construct a diagnostic model for pediatric migraine. **D** Receiver operating characteristic curve (ROC) analysis for the diagnostic value of microbial biomarkers modeled by ridge regression, LEfSe and random forest. **E** Correlation heatmap among gut bacterial genera of migraine children. **F** Comparison of the relative abundances of gut bacterial genera regulating tryptophan metabolism was used with the Mann–Whitney test

To identify specific genera for diagnosing pediatric migraine, ridge regression, LEfSe analysis, and random forest analysis were used. Ridge regression analysis showed that seven genera, including Bacteroides, Eggerthella, Erysipelatoclostridium, Lachnoclostridium, Parasutterella, Sphingobacterium, and Sutterella were selected as a combined indicator in diagnosing pediatric migraine, with an AUC of 0.973, a sensitivity of 97.6%, and a specificity of 90.9% (Fig. 3D1). Twenty-eight specific genera were identified by LEfSe analysis (Fig. 3A) as a combined indicator for diagnosing pediatric migraine with an AUC of 0.988, a sensitivity of 100%, and a specificity of 93.9% (Fig. 3D2). Nineteen genera with the highest model-building AUC value, validated by the AUC validation method (Fig. 3B), were selected as a combined indicator in diagnosing pediatric migraine by random forest analysis (Fig. 3C), with an AUC of 0.994, sensitivity of 97.6%, and specificity of 97.0% (Fig. 3D3). Then, the difference between the AUCs of the three methods was compared with the Hanley and McNeil methods, showing no significant difference among the AUCs of the three models. Therefore, the three models all have excellent potential value and exceptionally high sensitivity and specificity in diagnosing pediatric migraine. However, the combined indicator remodeled by ridge regression only includes seven genera, which is easier to apply (Supplementary Table 1).

#### Association among gut bacterial genera of migraine children

To further evaluate the relationship among genera in migraine children, Spearman correlation analysis was performed. The findings revealed that there were significantly strong correlations between Anaerococcus and Peptoniphilus (r = 0.948, *p* < 0.001), Peptoniphilus and Varibaculum (r = 0.912, *p* < 0.001), and Finegoldia and Tyzzerella (r = 0.900, *p* < 0.001) (Fig. 3E).

#### Alterations of gut microbiota regulating tryptophan metabolism

From the above results, it is clear that the gut microbiota may be involved in the pathogenesis of pediatric migraine. To investigate whether gut microbiota dysbiosis in pediatric migraine affects tryptophan metabolism, we further compared the differences in the relative abundance of eighteen genera involved in tryptophan metabolism between migraine children and healthy children [12–16]. We identified seventeen genera, such as Bacteroides and Sutterella, that showed significant differential relative abundance between the two groups (Fig. 3F).

### Tryptophan metabolism disturbance in pediatric migraine

#### Study population

Plasma samples were collected from a total of 51 migraine children (31 boys, mean age 10.75 ± 2.20 years), along with 120 healthy children (68 boys, mean age 9.91 ± 5.33 years). The mean age and sex of the migraine group and the healthy group did not differ significantly. The characteristics of pediatric migraine in our study are shown in Table 2. The course of pediatric migraine varied from 1 week to 72 months, with 32 children experiencing migraines lasting more than 3 months. The duration of pediatric migraine ranged from 2 to 48 h, with most migraines lasting between 2-8 h. More than half of children with migraine have more than 15 migraine attacks per month. The VAS assessment of pediatric migraine indicated moderate to severe pain in approximately 80% of children with migraines. 33 migraine children were in the ictal phase at the time of blood collection, and 11 migraine children experienced aura. 8 migraine children experienced visual aura, while 4 migraine children experienced sensory aura. Most children suffer from migraine without aura. And in pediatric migraine, aura symptoms are dominated by visual aura, such as black haze and flashes of light. Migraine in children primarily affects the frontal and bilateral temporal areas, whereas migraine in adults occurs primarily in the unilateral temporal region. (Table 2).

**Table 2:** Demographic and Clinical Characteristics of participants in the study of tryptophan metabolism

Demographic	Migraine	Healthy	*p*
Number	51	120	
Age (years)	10.75 ± 2.20^a^	9.91 ± 5.33^a^	0.280
Gender (male)	31(61%)	68(57%)	0.460
**Clinical characteristics**			
Course (months)	6.00 ± 23.00^b^		
Duration attacks (hours)	3.00 ± 4.00^b^		
VAS score	6.00 ± 2.00^b^		
Frequency (times/month)	18.00 ± 18.00^b^		
Ictal phase	33(65%)		
Aura	11(22%)		
**Aura**			
Visual aura	8(16%)		
Sensory aura	4(8%)		
**Concomitant symptoms**			
Nausea	33(65%)		
Vomiting	18(35%)		
Photophobia	20(39%)		
Phonophobia	20(39%)		
**Pain site**			
Unilateral temporal	7(14%)		
Bilateral temporal	16(31%)		
Frontal	25(49%)		
Parietal	10(20%)		
Occipital	9(18%)		

*VAS* visual analog scale

^a^normally distributed data, represented by Mean ± SD

^b^nonnormally distributed data, represented by Median ± IQR

#### Disturbance of tryptophan metabolism in pediatric migraine

**Fig. 4:**
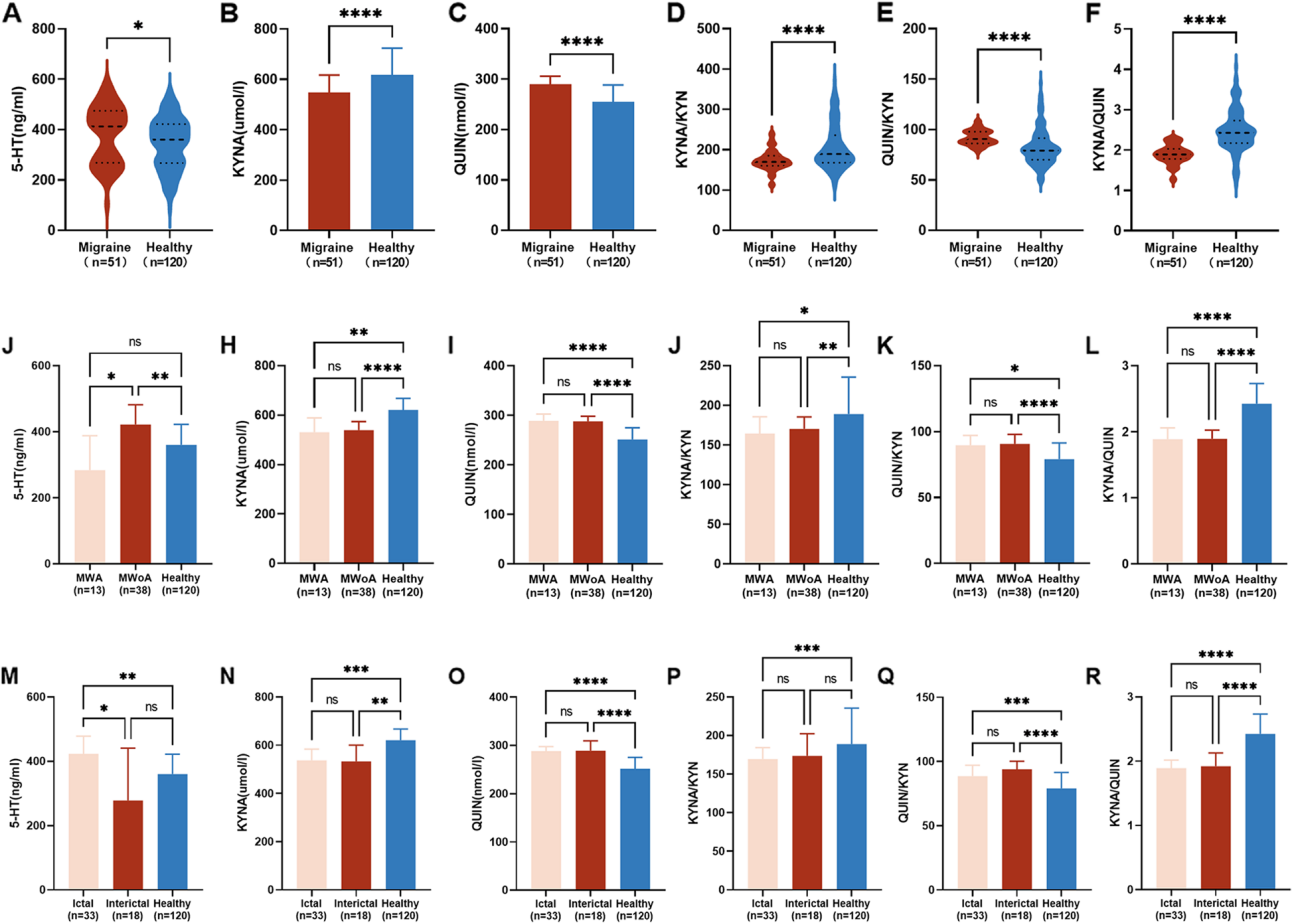
Tryptophan metabolites plasma levels in different subgroups of migraine and healthy children. Comparison of plasma levels of serotonin (5-HT), the ratio of kynurenic acid to kynurenine (KYNA/KYN), quinolinic acid to kynurenine (QUIN/KYN), kynurenic acid to quinolinic acid (KYNA/QUIN) in the migraine and healthy groups was used with Mann–Whitney test. Comparison of plasma levels of kynurenic acid (KYNA) and quinolinic acid (QUIN) was used with t test (**A-F**). Comparison of plasma levels of serotonin (5-HT), kynurenic acid (KYNA), quinolinic acid (QUIN) and the ratio of kynurenic acid to kynurenine (KYNA/KYN), quinolinic acid to kynurenine (QUIN/KYN), kynurenic acid to quinolinic acid (KYNA/QUIN) in healthy groups, MWA (migraine with aura), and MWoA (migraine without aura) was used Welch ANOVA test (**G-L**). Comparison of plasma levels of serotonin (5-HT), kynurenic acid (KYNA), quinolinic acid (QUIN) and the ratio of kynurenic acid to kynurenine (KYNA/KYN), quinolinic acid to kynurenine (QUIN/KYN), and kynurenic acid to quinolinic acid (KYNA/QUIN) in healthy groups and ictal and interictal groups was used Welch ANOVA test (**M-R**)

We investigated the plasma concentrations of TRP, 5-HT, KYN, KYNA, and QUIN, as well as the ratios of KYN/TRP, KYNA/KYN, QUIN/KYN, and KYNA/QUIN, in children with migraine and healthy children. Interestingly, children with migraine exhibited a trend of increases in plasma concentrations of 5-HT in the migraine group (5-HT(m) = 412.45 ng/ml, 5-HT(h) = 359.77 ng/ml, *p* = 0.0479) (Fig. 4A). Significant decreases in plasma concentrations of KYNA (KYNA(m) = 546.81 µmol/l, KYNA(h) = 617.63 µmol/l, *p* < 0.0001) (Fig. 4B) and significant increases in QUIN (QUIN(m) = 289.51 nmol/l, QUIN(h) = 254.39 nmol/l, *p* < 0.0001) (Fig. 4C) were observed in migraine children. Additionally, we observed a lower KYNA/KYN ratio (KYNA/KYN(m) = 169.61, KYNA/KYN(h) = 189.01, *p* < 0.0001) and a higher QUIN/KYN ratio in the migraine group than in the healthy group (QUIN/KYN(m) = 90.56, QUIN/KYN(h) = 79.14, *p* < 0.0001) (Fig. 4D, E). The KYNA/QUIN ratio was also significantly decreased in the children with migraine (KYNA/QUIN(m) = 1.89, KYNA/QUIN(h) = 2.43, *p* < 0.0001) (Fig. 4F). However, no significant differences were observed in the concentrations of TRP, KYN, and KYN/TRP ratio between the two groups. We also compare the difference in tryptophan metabolites between migraine and healthy children in different sex groups (Supplementary Fig. 2), which indicates that the difference in the plasma levels of KYNA, QUIN, KYNA/KYN ratio, QUIN/KYN ratio, and KYNA/QUIN ratio was not affected by sex. However, no significant difference in the 5-HT levels between the two groups was found in the male group. Similarly, we did not find alterations in the concentrations of TRP, KYN, and KYN/TRP ratio in migraine children compared with healthy children, either in the male or female group.

#### Disturbance of tryptophan metabolism in pediatric migraine with aura

To investigate whether TRP metabolites contribute to the occurrence of aura symptoms, we compared the plasma tryptophan metabolite concentrations among the migraine with aura group (MWA), migraine without aura group (MWoA), and healthy group. The plasma concentration of 5-HT was significantly higher in the MWoA group than in the MWA and healthy groups, and there was a decreased tendency of 5-HT in MWA than in healthy children (5-HT(MWoA) = 421.44 ng/ml, 5-HT(MWA) = 283.06 ng/ml, 5-HT(h) = 359.77 ng/ml, *p* = 0.003) (Fig. 4J). This suggests that a low concentration of plasma 5-HT may induce aura in migraine. The plasma concentrations of KYNA significantly decreased and QUIAN significantly increased in both the MWA and MWoA groups compared with the healthy group (KYNA(MWA) = 535.81 µmol/l, KYNA(MWoA) = 538.36 µmol/l, KYNA(h) = 620.59 µmol/l, *p* < 0.0001; QUIN(MWA) = 292.93 nmol/l, QUIN(MWoA) = 288.06 nmol/l, QUIN(h) = 251.49 nmol/l, *p* < 0.0001) (Fig. 4H, I). Additionally, the KYNA/KYN and QUIN/KYN ratios were significantly lower and higher in both the MWoA and MWA groups compared to the healthy group (KYNA/KYN(MWA) = 164.28, KYNA/KYN (MWoA) = 170.37, KYNA/KYN (h) = 189.01, *p* = 0.001; QUIN/KYN(MWA) = 89.56, QUIN/KYN(MWoA) = 90.69, QUIN/KYN(h) = 79.14, *p* < 0.0001) (Fig. 4J, K). The KYNA/QUIN ratio decreased significantly in both the MWoA and MWA groups compared to the healthy group (KYNA/QUIN(MWA) = 1.89, KYNA/QUIN(MWoA) = 1.90, KYNA/QUIN (h) = 2.43, *p* < 0.0001) (Fig. 4L). However, no significant differences were found in the concentrations of TRP, KYN, and the KYN/TRP ratio between MWA, MWoA, and the healthy group.

#### Disturbance of tryptophan metabolism in pediatric migraine in the ictal period

To investigate the potential role of TRP metabolites in triggering migraine attacks, we analyzed the plasma tryptophan metabolites in children with migraine during the ictal period and interictal period and in healthy children. The results showed significantly higher concentrations of 5-HT in migraine children during the ictal period compared to the interictal period and the healthy group (5-HT (ictal) = 422.61 ng/ml, 5-HT (interictal) = 277.63 ng/ml, 5-HT(h) = 359.77 ng/ml, *p* = 0.001) (Fig. 4M), suggesting that a sudden increase in 5-HT release may be a part of the triggering events that culminate in migraine attacks. The concentrations of KYNA were significantly lower in both the ictal period and interictal period groups than in the healthy group (KYNA (ictal) = 539.05 µmol/l, KYNA (interictal) = 536.28 µmol/l, KYNA (h) = 620.59 µmol/l, *p* < 0.0001) (Fig. 4N). In contrast, the concentrations of QUIN were significantly higher in the two groups than in the healthy group (QUIN (ictal) = 289.04 nmol/l, QUIN (interictal) = 288.20 nmol/l, QUIN (h) = 251.49 nmol/l, *p* < 0.0001) (Fig. 4O). Furthermore, both the ictal and interictal periods of the migraine group exhibited significantly lower KYNA/KYN and higher levels of QUIN/KYN compared to the healthy group (KYNA/KYN (ictal) = 169.51, KYNA/KYN (interictal) = 173.62, KYNA/KYN(h) = 189.01 *p* = 0.001; QUIN/KYN (ictal) = 88.61, QUIN/KYN (interictal) = 93.98, QUIN/KYN(h) = 79.14, *p* < 0.0001) (Fig. 4P, Q). KYNA/QUIN decreased significantly in the two groups compared to the healthy group (KYNA/QUIN (ictal) = 1.89, KYNA/QUIN (interictal) = 1.92, KYNA/QUIN (h) = 2.43, *p* < 0.001) (Fig. 4R). There were no significant differences in the concentrations of TRP, KYN, and the KYN/TRP ratio among the three groups.

#### Diagnostic value of tryptophan metabolites in pediatric migraine

**Fig. 5:**
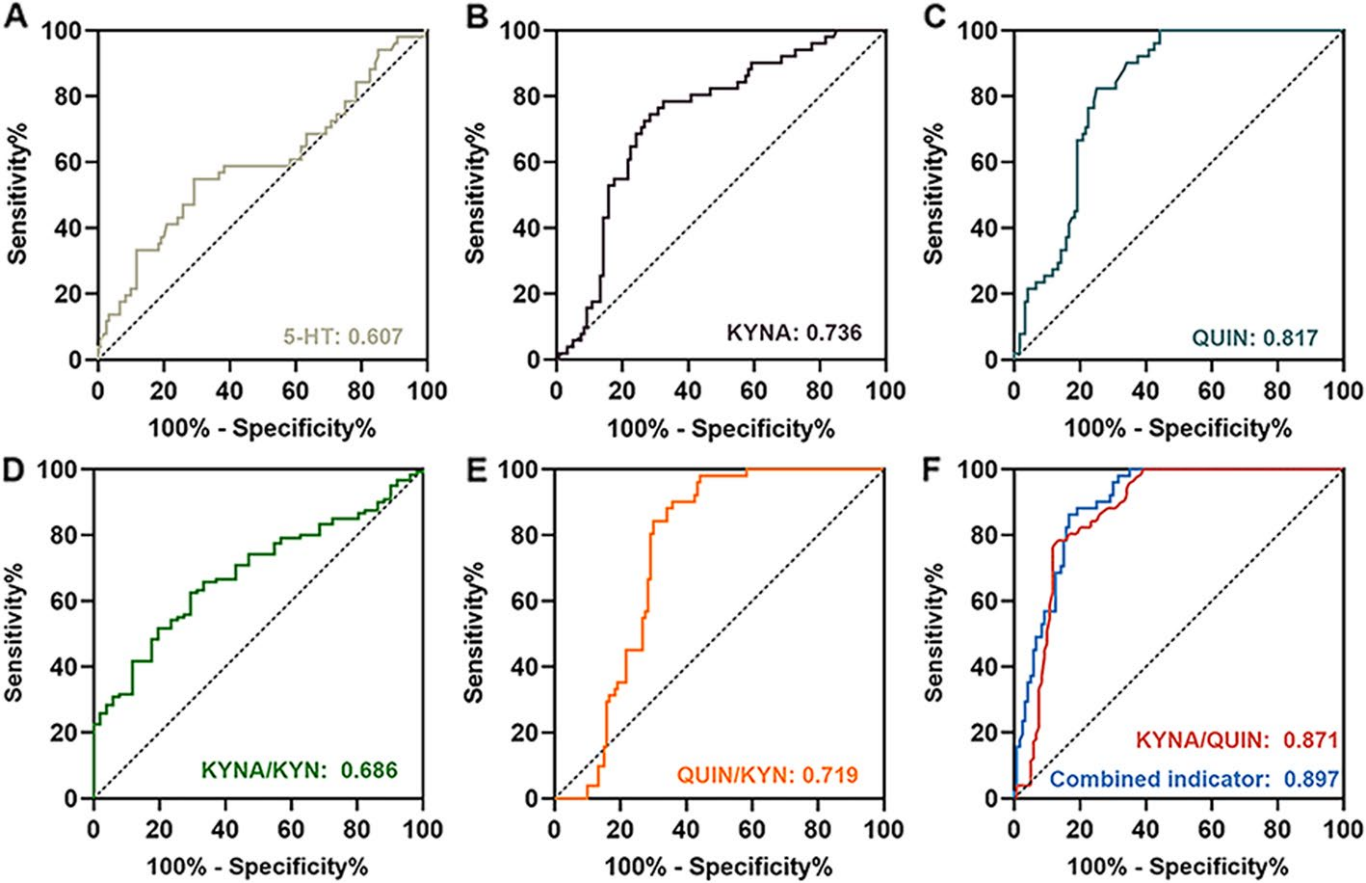
ROC analysis of the diagnostic value of tryptophan metabolite biomarkers. Receiver operating characteristic curve (ROC) of serotonin (5-HT), kynurenic acid (KYNA), quinolinic acid (QUIN) and the ratio of kynurenic acid to kynurenine (KYNA/KYN), quinolinic acid to kynurenine (QUIN/KYN), and kynurenic acid to quinolinic acid (KYNA/QUIN) for diagnosing pediatric migraine (**A-F**)

The diagnostic value of tryptophan metabolites was evaluated using ROC analysis (Supplementary Table 2) (Fig. 5). Among the eight indicators, KYNA/QUIN exhibited the highest diagnostic value for pediatric migraine (AUC: 0.871, 95% CI: 0.818–0.924), with a sensitivity of 86.7% and specificity of 78.4%. Additionally, QUIN also demonstrated good diagnostic ability for diagnosing migraine in children (AUC: 0.817, 95% CI: 0.756–0.879), with a sensitivity of 82.4% and a specificity of 75.0%. To further improve the diagnostic accuracy, we incorporated the biomarkers KYNA, QUIN, and 5-HT, which showed promising diagnostic abilities for pediatric migraine, into a combined indicator. Notably, the diagnostic performance of the biomarkers significantly improved in this combined indicator (AUC: 0.897, 95% CI: 0.851–0.942), with a sensitivity of 86.3% and specificity of 83.3%. A comparison of the AUCs between the KYNA/QUIN ratio and the combined indicator using the Hanley and McNeil methods revealed no significant difference in their diagnostic efficacy. Given the inclusion of fewer biomarkers in the KYNA/QUIN diagnostic model, we believe that KYNA/QUIN is more suitable as an excellent diagnostic indicator for pediatric migraine.

#### Risk and protective metabolites for pediatric migraine

**Fig. 6:**
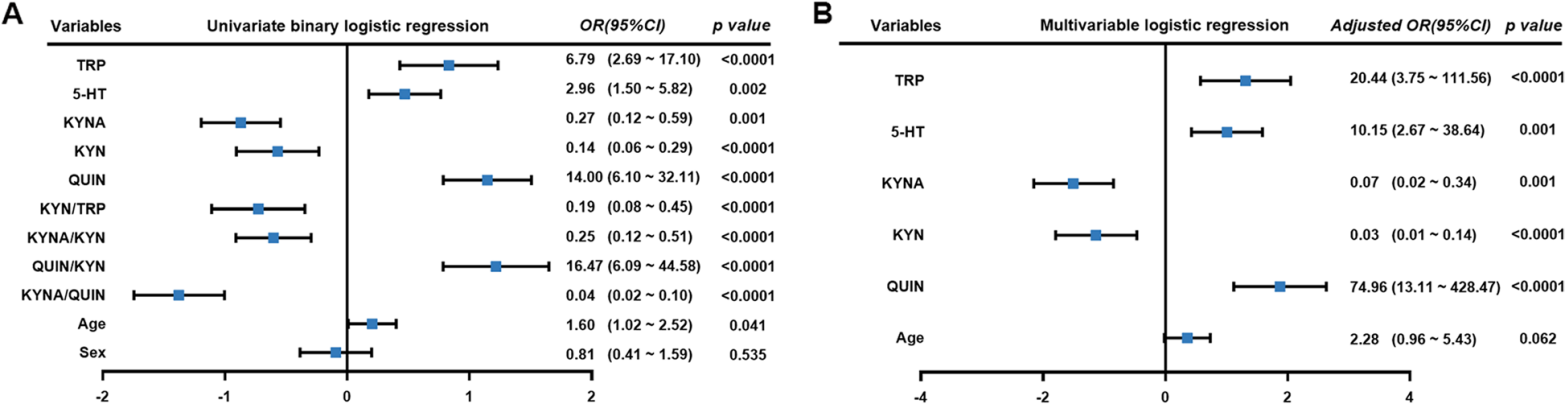
Logistic regression analysis of the association between tryptophan metabolites and pediatric migraine. The forest plot depicts log odds ratios (ORs) for tryptophan metabolites. On the right side, raw ORs and 95% CI for tryptophan metabolites are presented. **A** The OR values and 95% CI obtained from univariate binary logistic regression are displayed. Kynurenic acid (KYNA), kynurenine (KYN), the ratio of kynurenine to tryptophan (KYN/TRP), the ratio of kynurenic acid to kynurenine (KYNA/KYN), and the ratio of kynurenic acid to quinolinic acid (KYNA/QUIN) emerge as protective factors against pediatric migraine. Conversely, tryptophan (TRP), serotonin (5-HT), quinolinic acid (QUIN), the ratio of quinolinic acid to kynurenine (QUIN/KYN), and age are identified as risk factors for pediatric migraine. Sex may not be significantly associated with pediatric migraine. **B** The OR values and 95% CI for tryptophan metabolites from multivariable logistic regression reveal that tryptophan (TRP), quinolinic acid (QUIN), and serotonin (5-HT) are risk factors for pediatric migraine, while kynurenine (KYN), kynurenic acid (KYNA), and quinolinic acid (QUIN) serve as protective factors. Age may not be significantly associated with pediatric migraine

To identify the risk and protective metabolites associated with pediatric migraine, we categorized TRP, 5-HT, KYN, KYNA, and QUIN into low- and high-concentration groups and KYN/TRP, KYNA/KYN, QUIN/KYN, and KYNA/QUIN into low- and high-ratio groups using cutoff values (Supplementary Table 2). Additionally, all participants were divided into three groups according to whether they were ≤ 6 years old or > 12 years old, which were then converted into dummy variables. Subsequently, the impact of eleven indicators (TRP, 5-HT, KYN, KYNA, QUIN, KYN/TRP, KYNA/KYN, QUIN/KYN, KYNA/QUIN, age, sex) on pediatric migraine was assessed using univariate binary logistic regression. The findings revealed that KYN, KYNA, KYN/TRP, and KYNA/QUIN were protective factors for children with migraine. Conversely, TRP, 5-HT, QUIN, QUIN/KYN, and age were identified as risk factors for migraine in children (Fig. 6A). Subsequently, candidate variables with a p value < 0.05 from the univariate analysis were included in the multivariable model. The adjusted logistic regression analysis revealed that KYNA and KYN served as protective factors for children with migraine, while TRP, 5-HT, and QUIN were identified as risk factors for children with migraine (Fig. 6B).

#### Reference intervals for plasma concentrations of tryptophan metabolites

To aid in the diagnosis and prognosis assessment of pediatric migraine, we determined the normal reference intervals for plasma concentrations of tryptophan metabolites in children. The Kolmogorov‒Smirnov test was conducted on a cohort of 120 healthy children, revealing a skewed distribution for TRP and KYN data, while 5-HT, KYNA, and QUIN data exhibited a normal distribution. The 95% reference intervals for tryptophan metabolite levels in children are presented in Table 3. Furthermore, we examined the gender-based differences in the 95% reference intervals for tryptophan metabolites in children, as shown in Table 4. No statistically significant differences were observed in the plasma concentrations of TRP, KYN, 5-HT, KYNA, and QUIN between males and females.

**Table 3:** Normal reference intervals for plasma concentrations of tryptophan metabolites

Variables	Mean/Median(*n* = 120)	Lower limit	Upper limit
TRP	451.90^b^	266.48^b^	720.79^b^
5-HT	343.87^a^	325.42^a^	362.33^a^
KYN	3.30^b^	1.31^b^	4.25^b^
KYNA	617.63^a^	598.76^a^	636.50^a^
QUIN	254.39^a^	248.30^a^	260.48^a^

^a^normally distributed data, represented by Mean (95% CI);

^b^nonnormally distributed data, represented by Median (P2.5, P97.5)

**Table 4:** Comparison of plasma concentrations of tryptophan metabolite by sex

Variables	Male(*n* = 68)	Female(*n* = 52)	*p*
TRP	451.65(268.19,703.87)^b^	450.76(417.80,483.73)^a^	0.624
5-HT	342.87(317.47, 368.28)^a^	345.18(318.24, 372.12)^a^	0.904
KYN	3.31(1.30,4.35)^b^	3.29(1.09, 4.73)^b^	0.335
KYNA	616.48(592.90, 640.06)^a^	619.14(588.11, 650.16)^a^	0.892
QUIN	249.36(241.22, 257.49)^a^	260.97(252.01, 269.92)^a^	0.064

^a^normally distributed data, represented by $\overline{{\text{x}} }\pm 1.96 {\text{s}}$;

^b^nonnormally distributed data, represented by Median (P2.5, P97.5)

## Discussion

Based on our study on the role of gut microbiota and tryptophan metabolites in pediatric migraine, we demonstrated the presence of gut microbiota dysbiosis in children with migraine, particularly the gut microbiota involved in regulating tryptophan metabolism. We further investigated and confirmed dysregulated expression of tryptophan metabolites in migraine children. Our findings may support the hypothesis that the gut microbiota can modulate the development of pediatric migraine by regulating tryptophan metabolism. Moreover, we found that both gut microbiota and tryptophan metabolites exhibited significant diagnostic ability in pediatric migraine. These findings shed new light on the role of the gut-brain axis in the pathogenesis of pediatric migraine.

The analysis of alpha diversity revealed a significant difference in microbial richness and diversity between migraine children and healthy children, indicating a reduction in the richness and diversity of gut microbiota in pediatric migraine. Similarly, the beta diversity analysis also demonstrated a notable distinction between migraine children and healthy children, suggesting a significant difference in the composition of gut microbiota between the two groups. Additionally, we identified a total of twenty-five bacterial genera that exhibited distinctive abundance patterns between the two groups, suggesting an association between pediatric migraine and alterations in gut microbiota composition. It is worth noting that few studies have specifically focused on changes in the gut microbiota of children with migraine. In line with previous studies by Weiqing Jiang et al., significant beta diversity differences and variations in twenty-three genera were found between adults with migraine and healthy adults [17]. However, their study reported higher alpha diversity in adults with migraine, which contrasts with our findings. The microbiota composition can differ significantly between migraine children and adults due to variations in lifestyle, dietary habits, hormonal fluctuations, and other factors. The response of the gut microbiome to migraine can exhibit considerable variability. The complex interplay of genetics, environment, and host factors adds layers of complexity to microbiome studies, making direct comparisons challenging. The observed differences in alpha diversity between our study and that of Weiqing Jiang et al. highlight the intricate nature of the gut microbiota and emphasize the need for further research to identify the factors contributing to such differences. In addition, due to the lack of additional medical information on participants in the gut microbiota study, it cannot be ruled out that there may be other confounding factors that cause gut microbiota changes in migraine children.

Beyond alterations in richness and diversity, increased abundances of Proteobacteria were observed in the majority of the migraine children in our study. Proteobacteria is recognized as an indicator of microbial dysbiosis. The increase in Proteobacteria levels in migraine children might further support the opinion that microbial dysbiosis is closely associated with the pathogenesis of pediatric migraine. Studies have found that there is a significant increase in Proteus in the feces of people with a high-fat diet, so it is speculated that the excessive growth of Proteus may be related to a high-fat diet [18]. The high-fat diet-induced increase in the abundance of the phylum Proteobacteria was attributed to the increased bioavailability of respiratory electron acceptors of the host, such as oxygen and nitrate, which fueled the phylum Proteobacteria proliferation [18]. Therefore, a low-fat diet may be a good lifestyle to prevent migraines.

Furthermore, we employed a multiple analytical strategy in developing microbial diagnostic models for pediatric migraine, aiming to enhance the reliability of our models. We then evaluated the diagnostic performance of multiple microbial models, allowing us to identify the most effective among them. Our investigation identified a combined indicator comprising seven distinct genera, which demonstrate remarkable diagnostic capabilities, high sensitivity and specificity for diagnosing pediatric migraine. This remarkable diagnostic accuracy emphasizes the potential clinical utility of our microbial model in diagnosing pediatric migraine. Furthermore, our findings emphasize the importance of stool as a noninvasive diagnostic method for pediatric migraine. This realization sheds new light on future research and may change the diagnosis of pediatric migraine. Nonetheless, it is important to recognize that additional validation studies and rigorous testing in diverse populations are necessary to ensure the robustness and generalizability of the model.

It has been proposed that the tryptophan-kynurenine pathway may be one of the main mechanisms of the gut-brain axis, as the gut microbiota may alter the activities of synthase activity in bacterial strains of the intestine that can produce tryptophan metabolites, such as tryptophan, serotonin, quinolinic acid, and kynurenic acid, which can affect brain functions [19]. Therefore, we next investigated whether the composition of gut microbiota involving the regulation of tryptophan metabolism was altered in children with migraine. Our findings demonstrated that seventeen genera that have an impact on tryptophan metabolism showed a significant differential relative abundance between migraine children and healthy children. This observation may support the hypothesis that the gut microbiota may affect pediatric migraine by directly or indirectly regulating tryptophan metabolism. The limitation of this hypothesis arises from the fact that data on gut microbiota were collected from databases, but the studies did not investigate the concentrations of plasma tryptophan metabolites in those participants, which prevented us from directly verifying the association between gut microbiota and tryptophan metabolites.

However, it is important to highlight the new perspective that tryptophan metabolites may be involved in pediatric migraine pathogenesis. Therefore, we next focused our study on the important role of tryptophan metabolites in pediatric migraine by examining the plasma concentration of tryptophan metabolites. As expected, impaired tryptophan metabolism was found in children with migraine, as we observed a significant decrease in the plasma concentrations of KYNA, while 5-HT and QUIN showed a significant increase in migraine children compared to healthy children. These findings are in line with previous studies by Martina Curto et al., which reported decreased concentrations of KYNA in adults with chronic migraine or cluster headaches. KYNA has been shown to prevent trigeminovascular activation and cortical spreading depression in animal models of migraine [20, 21]. KYNA also interacts with endogenous N-methyl-D-aspartic acid (NMDA) receptors to inhibit the glutamate pathway, potentially preventing migraine-causing sensitization processes [22]. Our study also found an increased 5-HT plasma concentration in migraine in the ictal period, which is consistent with previous studies [23], indicating that a sudden increase in 5-HT release may be a triggering event that culminates in migraine attacks or a response elevation to the migraine attack. QUIN is known as a pro-oxidant inflammatory molecule that activates NMDA receptors and regulates glutamate release or uptake to facilitate migraine progression [24], which could explain the higher concentrations of QUIN observed in migraine children in our study. Although some studies have reported changed plasma concentrations of TRP and KYN in adults with migraine [25], we did not find a significant difference in their plasma concentrations in migraine children.

In addition, we are the first to evaluate KYN/TRP, KYNA/KYN, and QUIN/KYN in pediatric migraine, which may reflect the activity of enzymes in the KP. Surprisingly, we found a significant decrease in KYNA/KYN, suggesting a reduction in the conversion of KYN to KYNA, a process catalyzed by kynurenine aminotransferase (KAT). In our study, a significantly higher QUIN/KYN ratio was also observed in migraine children, suggesting an increased conversion of KYN to QUINA, which is facilitated by some enzymes, such as kynurenine 3-monooxygenase (KMO), kynurenine lyase (KYNU), and 3-hydroxy anthranilate dioxygenase (HAOO). Based on these findings, we believe that the dysregulated activity of enzymes in the KP may be involved in the pathogenesis of pediatric migraine. Supporting this idea, upregulated mRNA levels of these enzymes (KMO, KYNU, and HAOO) were found at the spinal cord level in a chronic constriction injury rat model, confirming the association between the development of neuropathic pain and the activity of KP pathway enzymes [26]. We also found that KYNA/QUIN was significantly lower in migraine children, indicating that the imbalance between the neuroprotective and neurotoxic properties of KYNA and QUIN may lead to the progression of pediatric migraine. A previous study also observed that KYNA/QUIN decreased in the plasma of depressive patients [27]. Moreover, our study identified the risk factors and protective factors in TRP metabolites for pediatric migraine, showing that KYN, KYNA, KYN/TRP, KYNA/KYN, and KYNA/QUIN play a protective role in pediatric migraine, and TRP, 5-HT, QUIN, QUIN/KYN and age play a risk role in pediatric migraine. These findings further support the idea that TRP metabolites may play a key role in the development of pediatric migraine.

We further evaluated the diagnostic performance of TRP metabolites in pediatric migraine. The results indicated that KYNA/QUIN was an excellent diagnostic indicator for pediatric migraine, which provides the possibility of using a rapid and quantitative marker to aid in the accurate diagnosis and assessment of migraine in children. By utilizing such quantitative indicators, clinicians can potentially facilitate timely intervention, ultimately improving the quality of care provided to migraine children. Furthermore, our study extends its contributions to the establishment of normal reference intervals for plasma concentrations of tryptophan metabolites in children. This step is pivotal for realizing clinical applications. By determining these reference intervals, we have provided a foundation for standardized clinical interpretation, ensuring that the diagnostic value of these metabolites can be effectively gauged in a pediatric context. It is important, however, to acknowledge the complexities inherent in translating research findings into practical clinical applications. Rigorous validation, further refinement, and extensive comparative studies are indispensable to ensure the reliability and generalizability of these diagnostic markers.

Moreover, previous studies suggested that KYN and KYNA may potentially play a protective role in pediatric migraine by inhibiting migraine-related peptidergic systems. In our previous study, we reported higher plasma concentrations of pituitary adenylate cyclase-activating polypeptide-38 (PACAP-38) and calcitonin gene-related peptide (CGRP) in children with migraine [28]. Kageneck et al. showed that KYN inhibits capsaicin-induced elevation of CGRP in the caudate nucleus of the trigeminal nerve [29]. Moreover, one study revealed that the expression of plasma PACAP-38 in a rat model decreased after KYNA treatment [30]. It could be hypothesized that the expression of TRP metabolites, particularly KYN and KYNA, may inhibit CGRP and PACAP-38 expression, potentially relieving pediatric migraine. Interestingly, there is also an intimate relationship between neuropeptides, such as CGRP, and gut microbiota. On the one hand, CGRP is believed to have an antimicrobial impact on various gut bacterial strains, such as Escherichia coli, Enterococcus faecalis, and Lactobacillus acidophilus. On the other hand, CGRP signaling could be influenced by autoantibodies governed by gut microbiota [31]. Theoretically, certain microorganisms may create neuropeptide-like compounds, and the gut microbiota will respond to neuropeptides if they express the appropriate receptors [32]. However, there is little direct evidence that these neuropeptides participate in communication between the gut microbial community and the central nervous system. It also remains to be investigated whether changes in the microbial ecology in the gut have an effect on neuropeptide systems in the central nervous system. Additionally, the exploration of the interrelationship between neuropeptides, tryptophan metabolites, and gut microbiota may provide a potential mechanism for elucidating the pathophysiology of pediatric migraine.

In conclusion, our study provides evidence of gut microbiota dysbiosis in migraine children, suggesting its potential involvement in the development of pediatric migraine. We identified a combined indicator of seven genera that demonstrates excellent diagnostic ability, high sensitivity, and specificity for diagnosing pediatric migraine. Interestingly, we observed alterations in the composition of gut microbiota involved in tryptophan metabolism in migraine children. We further found that plasma tryptophan metabolites were altered in migraine children. More importantly, we identified KYNA/QUIN as a diagnostic indicator with outstanding diagnostic ability for pediatric migraine. In summary, our study suggests that the gut microbiota can regulate tryptophan metabolism, particularly the kynurenine pathway, to be involved in the pathogenesis of pediatric migraine. These findings contribute to the understanding of the role of the gut-brain axis in migraine and provide a potential mechanism for further exploration of pediatric migraine.

### Supplementary Information

**Additional file 1: Supplementary fig. 1.** Differences in gut bacterial genera composition between migraine and healthy children of different sex groups. A. Comparison of the relative abundances of gut bacterial genera in healthy children and migraine children of the male group was used with the Mann-Whitney test. B. Comparison of the relative abundances of gut bacterial genera in healthy children and migraine children of the female group was used with the Mann-Whitney test.

**Additional file 2**: **Supplementary fig. 2.** Differences in tryptophan metabolite levels between migraine and healthy children of different sex groups. Comparison of plasma levels of serotonin (5-HT), the ratio of kynurenic acid to kynurenine (KYNA/KYN), quinolinic acid to kynurenine (QUIN/KYN), kynurenic acid to quinolinic acid (KYNA/QUIN) in the migraine and healthy children of the male group was used with Mann-Whitney test. Comparison of plasma levels of kynurenic acid (KYNA) and quinolinic acid (QUIN) in the migraine and healthy children of the male group was used with t test (A-F). Comparison of plasma levels of serotonin (5-HT), the ratio of kynurenic acid to kynurenine (KYNA/KYN), quinolinic acid to kynurenine (QUIN/KYN), kynurenic acid to quinolinic acid (KYNA/QUIN) in the migraine and healthy children of the female group was used with Mann-Whitney test. Comparison of plasma levels of kynurenic acid (KYNA) and quinolinic acid (QUIN) in the migraine and healthy children of the female group was used with t test (G-L).

**Additional file 3: Supplementary table 1.** ROC analysis of the diagnostic value of gut microbiota for pediatric migraine. **Supplementary table 2.** The diagnostic value of tryptophan metabolites for pediatric migraine.

## Data Availability

The datasets used or analyzed during the current study are available from the corresponding author on reasonable request.

## Abbreviations

Trp: Tryptophan
5-HT: Serotonin
KYN: Kynurenine
KYNA: Kynurenic acid
QUIN: Quinolinic acid
VAS: Visual analog scale
PCoA: Principal coordinates analysis
LEfSe: Linear discriminant analysis effect size
ROC: Receiver operating characteristic curve
MWA: Migraine with aura
MWoA: Migraine without aura
AUC: Area under the curve
KMO: 3-Monooxygenase
KYNU: Kynurenine lyase
HAOO: 3-Hydroxy anthranilate dioxygenase
PACAP-38: Pituitary adenylate cyclase-activating polypeptide-38
CGRP: Calcitonin gene-related peptide
