# Supplementary figures and images for: Tryptophan metabolites and gut microbiota play an important role in pediatric migraine diagnosis
Source: J Headache Pain. 2024 Jan 5;25(1):2. doi: 10.1186/s10194-023-01708-9 (PMC10768287; doi:10.1186/s10194-023-01708-9)

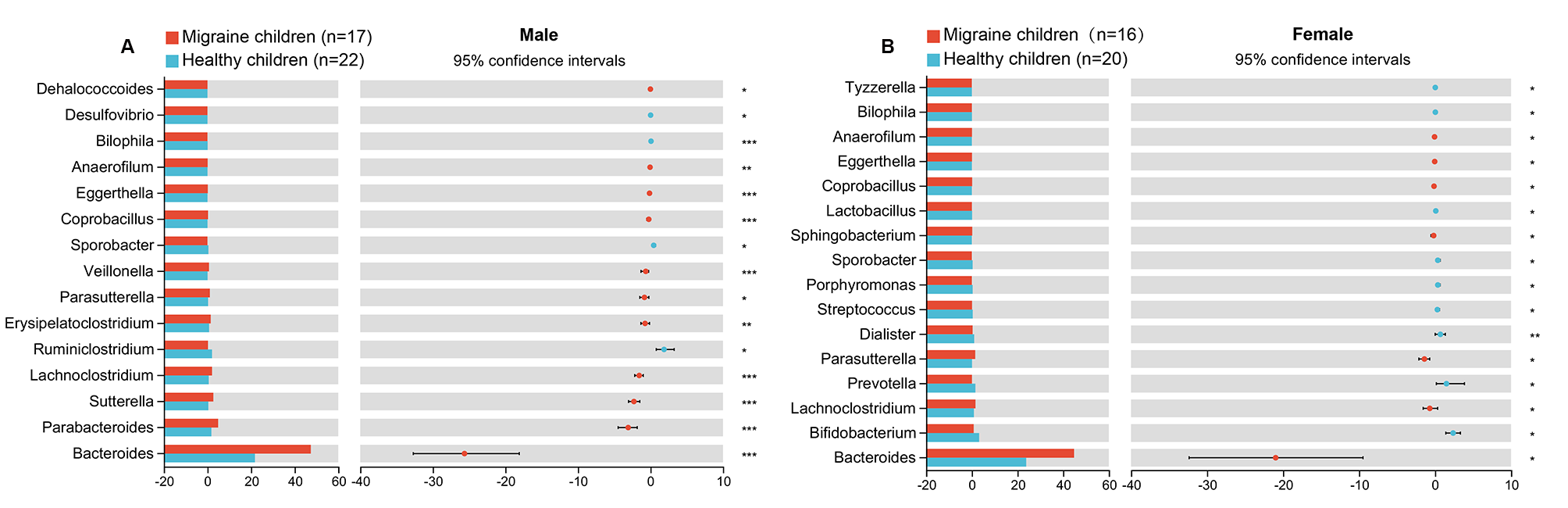

Supplement: Supplementary file 1 — Additional file 1: Supplementary fig. 1. Differences in gut bacterial genera composition between migraine and healthy children of different sex groups. A. Comparison of the relative abundances of gut bacterial genera in healthy children and migraine children of the male group was used with the Mann-Whitney test. B. Comparison of the relative abundances of gut bacterial genera in healthy children and migraine children of the female group was used with the Mann-Whitney test. [file 10194_2023_1708_MOESM1_ESM.tif]

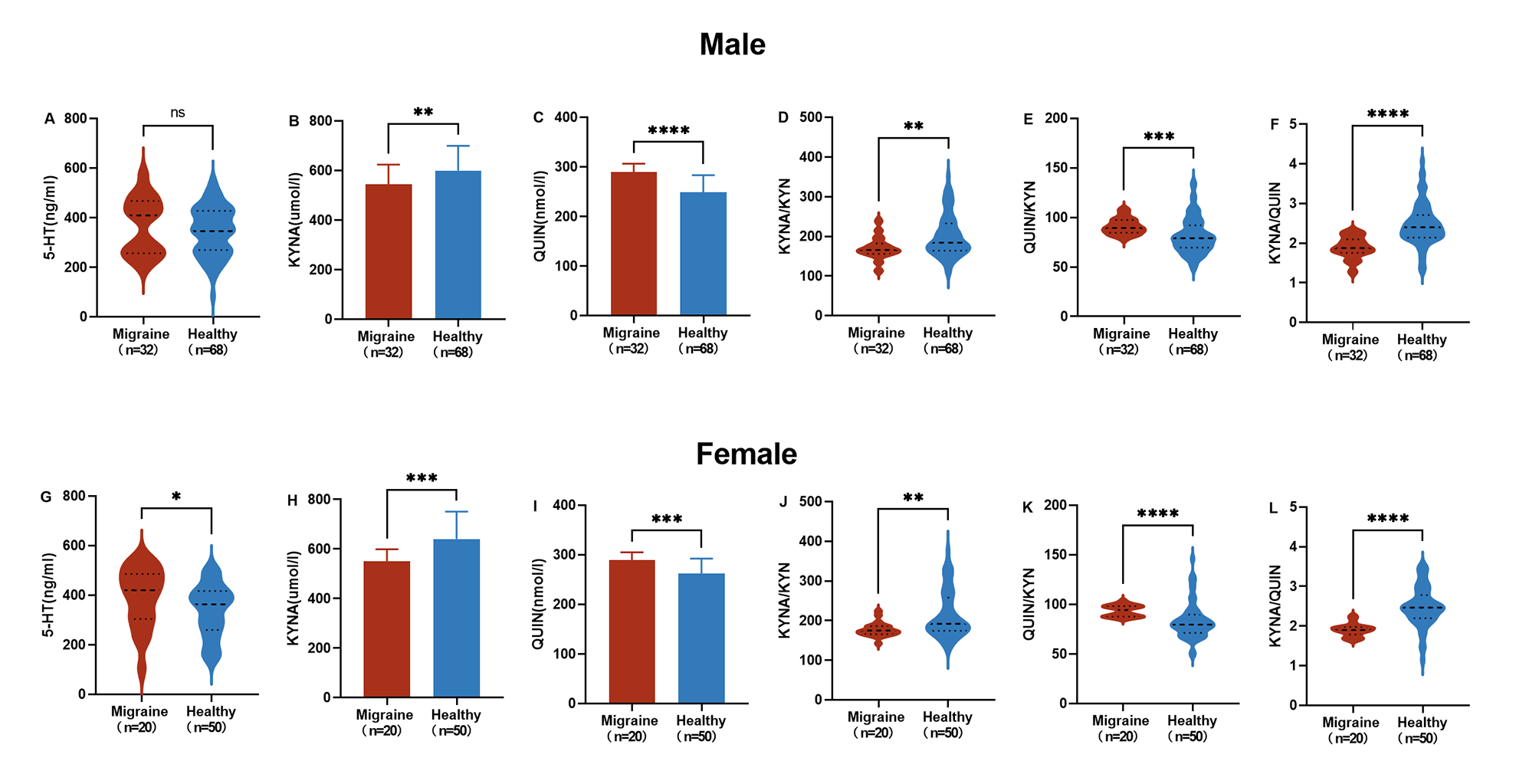

Supplement: Supplementary file 2 — Additional file 2: Supplementary fig. 2. Differences in tryptophan metabolite levels between migraine and healthy children of different sex groups. Comparison of plasma levels of serotonin (5-HT), the ratio of kynurenic acid to kynurenine (KYNA/KYN), quinolinic acid to kynurenine (QUIN/KYN), kynurenic acid to quinolinic acid (KYNA/QUIN) in the migraine and healthy children of the male group was used with Mann-Whitney test. Comparison of plasma levels of kynurenic acid (KYNA) and quinolinic acid (QUIN) in the migraine and healthy children of the male group was used with t test (A-F). Comparison of plasma levels of serotonin (5-HT), the ratio of kynurenic acid to kynurenine (KYNA/KYN), quinolinic acid to kynurenine (QUIN/KYN), kynurenic acid to quinolinic acid (KYNA/QUIN) in the migraine and healthy children of the female group was used with Mann-Whitney test. Comparison of plasma levels of kynurenic acid (KYNA) and quinolinic acid (QUIN) in the migraine and healthy children of the female group was used with t test (G-L). [file 10194_2023_1708_MOESM2_ESM.tif]
