# Supplementary material for: Tryptophan metabolites and gut microbiota play an important role in pediatric migraine diagnosis
Source: J Headache Pain. 2024 Jan 5;25(1):2. doi: 10.1186/s10194-023-01708-9 (PMC10768287; doi:10.1186/s10194-023-01708-9)
Supplement: Supplementary file 3 — Additional file 3: Supplementary table 1. ROC analysis of the diagnostic value of gut microbiota for pediatric migraine. Supplementary table 2. The diagnostic value of tryptophan metabolites for pediatric migraine. [file 10194_2023_1708_MOESM3_ESM.pdf]

Supplementary table 1. ROC analysis of the diagnostic value of gut microbiota for pediatric migraine

Variables	AUC	95% CI	Sensitivity	Specificity	Cutoff	Youden Index
Twenty-eight genera (LEfSe)	0.988	0.966-1.010	1.000	0.939	0.198	0.939
Nineteen genera (Random forest)	0.994	0.983-1.005	0.976	0.97	0.418	0.946
Seven genera (Logistic regression)	0.973	0.941-1.006	0.976	0.909	0.224	0.885

Supplementary table 2. The diagnostic value of tryptophan metabolites for pediatric migraine

Variables	AUC	95% CI	Sensitivity	Specificity	Cutoff	*p*
TRP	0.578	0.495-0.661	0.882	0.475	434.94	0.107
5-HT	0.596	0.497-0.694	0.549	0.708	408.93	0.048
KYN	0.543	0.459-0.627	0.475	0.804	3.33	0.373
KYNA	0.736	0.657-0.815	0.717	0.745	573.75	<0.0001
QUIN	0.817	0.756-0.879	0.824	0.750	274.64	<0.0001
KYN/TRP	0.576	0.491-0.661	0.458	0.863	1.54	0.117
KYNA/KYN	0.686	0.605-0.766	0.625	0.706	178.74	<0.0001
QUIN/KYN	0.749	0.678-0.820	0.902	0.642	82.67	<0.0001
KYNA/QUIN	0.871	0.818-0.924	0.867	0.784	2.05	<0.0001
Combined	0.897	0.851-0.942	0.863	0.833	0.31	<0.0001

Combined indicator: KYNA, QUIN, and 5-HT
